# Would procalcitonin measurement aid antimicrobial stewardship in a UK district general hospital mixed adult critical care population?

**DOI:** 10.1186/cc10637

**Published:** 2012-03-20

**Authors:** J Clayton, J White, L Wilson, M Leonard, J Cuniffe

**Affiliations:** 1 Wirral University Teaching Hospital NHS Foundation Trust, Wirral, UK

## Introduction

We sought to establish what impact knowledge of procalcitonin (PCT) levels could have on antimicrobial prescribing and stewardship within our 18-bed mixed critical care unit. Assicot and colleagues demonstrated that PCT levels are raised during sepsis and can correlate with the severity [1]. The PCT level peaks after 6 to 12 hours and has a half-life of approximately 25 to 36 hours in critically ill patients [2], declining with adequate treatment. A recent multicentre trial demonstrated reduced duration of antibiotic therapy by using PCT-guided treatment strategy; however, only 10% of the cohort was surgical patients and therefore this finding cannot be extrapolated to a general critical care population [3].

## Methods

The question was posed: would knowledge of PCT levels have altered real-time clinical management of patients on established antimicrobial therapy? Over a 2-month period patients were treated in a conventional manner based on clinical findings and standard investigations. Plasma samples from days 0 (respective to antimicrobial therapy) 1, 3, 5 and 7 were analysed for PCT. Nonparametric statistical analysis of PCT levels was available for a retrospective multidisciplinary team review of case notes. This was performed within the context of a local service review and the chair of the local ethics committee gave approval for analysis of plasma samples and case-note review.

## Results

Twenty-seven patients were identified. Antimicrobial cessation was deemed possible in seven of these cases at day 5. Nonescalation of treatment was supported in six further cases. In one case treatment had been escalated and PCT supported this decision. This would have resulted in 19 fewer days of antibiotic therapy.

## Conclusion

Our experience suggests the availability of the PCT response between days 0 and 5 would have been a useful adjunct in monitoring treatment of sepsis on our unit and would have facilitated timely de-escalation and hence exposure to antimicrobial therapy. We hypothesise such a reduction could help to prevent antimicrobial resistance, lead to decreased pharmacy and consumable costs and reduce the incidence of adverse antimicrobial-related events.
